# *Apolipoprotein E* E3/E4 genotype is associated with an increased risk of type 2 diabetes mellitus complicated with coronary artery disease

**DOI:** 10.1186/s12872-024-03831-0

**Published:** 2024-03-15

**Authors:** Wenhao Chen, Bin Li, Hao Wang, Guoliang Wei, Kehui Chen, Weihong Wang, Shen Wang, Yuanliang Liu

**Affiliations:** 1 Center for Cardiovascular Diseases, Meizhou People’s Hospital, Meizhou Academy of Medical Sciences, No. 63 Huangtang Road, Meijiang District, Meizhou, China; 2 Guangdong Provincial Engineering and Technology Research Center for Molecular Diagnostics of Cardiovascular Diseases, Meizhou People’s Hospital, Meizhou Academy of Medical Sciences, Meizhou, China; 3 Department of Computer Tomography, Meizhou People’s Hospital, Meizhou Academy of Medical Sciences, Meizhou, China

**Keywords:** Apolipoprotein E, Coronary artery disease, Diabetes mellitus, Polymorphism

## Abstract

**Objective:**

Dyslipidemia is a co-existing problem in patients with diabetes mellitus (DM) and coronary artery disease (CAD), and apolipoprotein E (APOE) plays an important role in lipid metabolism. However, the relationship between the *APOE* gene polymorphisms and the risk of developing CAD in type 2 DM (T2DM) patients remains controversial. The aim of this study was to assess this relationship and provide a reference for further risk assessment of CAD in T2DM patients.

**Methods:**

The study included 378 patients with T2DM complicated with CAD (T2DM + CAD) and 431 patients with T2DM alone in the case group, and 351 individuals without DM and CAD were set as controls. The *APOE* rs429358 and rs7412 polymorphisms were genotyped by polymerase chain reaction (PCR) - microarray. Differences in *APOE* genotypes and alleles between patients and controls were compared. Multiple logistic regression analysis was performed after adjusting for age, gender, body mass index (BMI), history of smoking, and history of drinking to access the relationship between *APOE* genotypes and T2DM + CAD risk.

**Results:**

The frequencies of the *APOE* ɛ3/ɛ4 genotype and ε4 allele were higher in the T2DM + CAD patients, and the frequencies of the *APOE* ɛ3/ɛ3 genotype and ε3 allele were lower than those in the controls (all *p* < 0.05). The T2DM + CAD patients with ɛ4 allele had higher level in low-density lipoprotein cholesterol (LDL-C) than those in patients with ɛ2 and ɛ3 allele (*p* < 0.05). The results of logistic regression analysis showed that age ≥ 60 years old, and BMI ≥ 24.0 kg/m^2^ were independent risk factors for T2DM and T2DM + CAD, and *APOE* ɛ3/ɛ4 genotype (adjusted odds ratio (OR) = 1.93, 95% confidence interval (CI) = 1.18–3.14, *p* = 0.008) and ɛ4 allele (adjusted OR = 1.97, 95% CI = 1.23–3.17) were independent risk factors for T2DM + CAD. However, the *APOE* genotypes and alleles were not found to have relationship with the risk of T2DM.

**Conclusions:**

*APOE* ε3/ε4 genotype and ε4 allele were independent risk factors for T2DM complicated with CAD, but not for T2DM.

## Introduction

Cardiovascular disease (CVD) is one of the major causes of disease burden and even death [1]. Coronary artery disease (CAD) is a kind of thrombotic cardiovascular disease, which is caused by vascular obstruction and coronary artery function changes mediated by coronary atherosclerosis, and then myocardial ischemia and hypoxia [2, 3]. CAD is one of the diseases that seriously affect human health at present, and its morbidity and mortality are on the rise in recent years [4, 5]. Diabetes mellitus (DM) is a disorder of glucose and lipid metabolism characterized by chronic hyperglycemia, which has become a serious global health problem [6]. About 90% of DM patients are type 2 diabetes mellitus (T2DM), which is non-insulin-dependent DM, its prevalence is high, onset occult, early symptoms are not obvious [7, 8]. The social and economic burden associated with T2DM remains significant in China [9].

CAD and DM are two chronic diseases which are seriously harmful to human health. DM patients are more prone to lipid metabolism disorders than non-DM patients, and the synergistic effect of the glucose and lipid metabolism disorders can further increase the risk of CAD in DM patients [10, 11]. DM can have a negative impact on existing CAD [12]. DM and CAD are both metabolic chronic diseases, they have common risk factors, and mutual risk factors, such as obesity, inflammation, oxidative stress, insulin resistance, and factors associated with increased microvascular and macrovascular damage [13]. When the two diseases co-exist, it will further accelerate the damage of tissues and organs, resulting in increased mortality and disability [14].

Both CAD and DM are associated with dyslipidemia, which may be due to the role of insulin resistance in lipid metabolism [15]. Lipid metabolism disorders are closely related to DM and CAD, and apolipoprotein plays an important role in lipid metabolism. Apolipoprotein E (ApoE) is a multifunctional glycoprotein. ApoE has the activity related to lipid metabolism and binding to lipoprotein. It mediates chylomicron endocytosis on the one hand and participates in catabolism of triacylglycerol on the other hand through corresponding receptors present on liver cells or peripheral cells [16]. ApoE is encoded by the *APOE* gene. The human *APOE* gene located on chromosome 19q13.2, is about 3.7 kb long and contains four exons and three introns [17]. Some sites of *APOE* gene are known to have single-nucleotide polymorphisms (SNPs), and there are differences in nucleotides corresponding to amino acids at 112 (rs429358) and 158 (rs7412) positions in the encoding ApoE peptide chain, forming three alleles of ε2, ε3 and ε4 [18], and six common *APOE* genotypes of ɛ2/ɛ2, ɛ2/ɛ3, ɛ2/ɛ4, ɛ3/ɛ3, ɛ3/ɛ4, and ɛ4/ɛ4 [19]. The *APOE* gene is one of the most important genetic factors in determining blood lipid levels, and ApoE encoded by different *APOE* genotypes shows a preference for and ability to bind to specific lipids [20].

Studies have showed that *APOE* gene polymorphism was established to be related to CAD and T2DM, respectively [21, 22]. *APOE* ε4 allele is associated with the increased risk of CAD [23, 24]. Furthermore, *APOE* allele ε4 is associated with the increased risk for the development of T2DM [25, 26]. However, the relationship between the *APOE* gene polymorphisms and the risk of developing CAD in T2DM patients (T2DM complicated with CAD patients) (T2DM + CAD) remains controversial. In the current study, we intended to explore the association between *APOE* gene polymorphisms and the risk of CAD in T2DM patients.

## Materials and methods

### Study participants

This study was a case-control study of the association between *APOE* gene polymorphism and the risk of developing CAD in T2DM patients. A total number of 1160 subjects were recruited from November 2019 to August 2023, included 378 patients with T2DM + CAD and 431 patients with T2DM in the case group, and 351 healthy individuals without DM and CAD were set as controls.

The diagnostic criteria of T2DM were the following: (1) There were typical clinical symptoms of diabetes (polydipsia, polydipsia, polyuria, polydipsia, unexplained weight loss), and random intravenous plasma glucose is ≥ 11.1mmol/L; or fasting blood glucose (FBG) is ≥ 7mmol/L; or blood glucose level at 2-hour oral glucose tolerance test is ≥ 11.1mmol/L [27]. The diagnostic criteria for CAD: Coronary angiography (CAG) showed that at least one of the main epicardial vessels (left main branch, anterior descending branch (diagonal branch), circumflex branch (blunt marginal branch), and right coronary artery) had a diameter stenosis > 50% and was clinically diagnosed as myocardial infarction [28, 29].

### Data Collection

Basic information was collected from our hospital’s medical record system, including age, gender, body mass index (BMI), history of smoking, and history of drinking. BMI was divided into three subgroups based on the Chinese criteria [30, 31]: <18.5 kg/m^2^, 18.5–23.9 kg/m^2^, and ≥ 24.0 kg/m^2^. Early morning fasting blood collection, serum separation. Lipid levels in serum samples were assessed using an automated biochemical analysis system (Olympus AU5400 system, Tokyo, Japan) with commercially available kits, include total cholesterol (TC), triglyceride (TG), high-density lipoprotein cholesterol (HDL-C), and low-density lipoprotein cholesterol (LDL-C) (Kefang Biotechnology Co., Ltd, Guangzhou, China) (ID: 20,172,400,246, 20,172,400,250, 20,172,400,249, and 20,172,400,252), Apolipoprotein A1 (Apo-A1), and Apolipoprotein B (ApoB) (Chuangyi Biochemical Engineering Co., Ltd, Zhongshan, China) (ID: 20,192,400,612, and 20,192,400,613).

### DNA extraction and genotyping

Genomic DNA was extracted from venous blood collected from EDTA anticoagulant collection vessels using a blood DNA isolation kit (Qiagen GmbH, Germany). The quality and concentration of the DNA were assessed using a Nano-Drop 2000™ spectrophotometer (ThermoFisher Scientific, Waltham, MA, USA). Genotyping of the *APOE* gene were amplified by PCR - microarray method (Sinochips Bioscience Co., Ltd., Zhuhai, China) (ID: 20,173,400,132). The PCR procedure was: 2 min at 50 ℃, 15 min at 95℃ for initial denaturation, and 45 thermal cycles (94℃ for 30 s and 65℃ for 45 s). The products amplified by PCR were added into the gene chip, the amplification products of the wild-type template were hybridized with the wild-type probes, and the amplification products of the mutant template were hybridized with the mutant probes, and the genotype of the sample was determined by the hybridization reaction signal.

### Statistical analysis

All statistical analysis were performed using SPSS statistical software version 26.0 (IBM Inc., USA). Continuous variables were expressed as means ± standard deviations and were compared using either Student’s t-test or the Mann-Whitney U test. Genotype composition ratios and allele frequencies between groups were analyzed with the *Chi*-square test. Hardy-Weinberg equilibrium in the patients and controls was evaluated by *Chi*-square test.

T2DM + CAD patients with the ɛ2/ɛ4 genotype (*n* = 12) were excluded from the analysis of the relationship between *APOE* alleles and clinical characteristics of patients because of the opposite effects of the ε2 and ε4 alleles in lipid metabolism [21, 24]. Logistic regression analysis was applied to examine the relationship between *APOE* gene polymorphisms and T2DM + CAD, and patients with T2DM, respectively. Univariate regression analysis was performed to obtain the unadjusted odds ratio (OR), and multiple logistic regression analysis was performed to obtain the adjusted OR. *p* < 0.05 was considered to represent statistical significance.

## Results

### Characteristics of subjects

The difference in age distribution among the three groups was statistically significant (*p* < 0.001). There were 94 (26.8%) cases with BMI < 18.5 kg/m^2^ and 60 (17.1%) cases with BMI ≥ 24.0 kg/m^2^ in controls, while 15 (3.5%) cases BMI < 18.5 kg/m^2^ and 199 (46.2%) cases with BMI ≥ 24.0 kg/m^2^ in T2DM patients, 19 (5.0%) cases with BMI < 18.5 kg/m^2^ and 160 (42.3%) cases with BMI ≥ 24.0 kg/m^2^ in T2DM + CAD patients. The difference in BMI distribution among the three groups was statistically significant (*p* < 0.001). The differences of TC (*p* < 0.001), TG (*p* < 0.001), HDL-C (*p* = 0.001), LDL-C (*p* = 0.023), Apo-A1 (*p* = 0.046) and Apo-B (*p* < 0.001) levels among the groups were statistically significant. There were not statistically significant differences in the percentage of subjects with a history of smoking (*p* = 0.376), and alcoholism (*p* = 0.243) (Table 1).

**Table 1:** Clinical characteristics of the subjects of this study

Variables	Total (*n* = 1160)	Controls (*n* = 351)	T2DM patients (*n* = 431)	T2DM complicated with CAD patients (*n* = 378)	p values
Age, years					
< 60, n(%)	497(42.8)	219(62.4)	177(41.1)	101(26.7)	< 0.001
≥ 60, n(%)	663(57.2)	132(37.6)	254(58.9)	277(73.3)	
Gender					
Male, n(%)	754(65.0)	226(64.4)	285(66.1)	243(64.3)	0.831
Female, n(%)	406(35.0)	125(35.6)	146(33.9)	135(35.7)	
BMI (kg/m^2^)					
< 18.5	128(11.0)	94(26.8)	15(3.5)	19(5.0)	< 0.001
18.5–23.9	613(52.8)	197(56.1)	217(50.3)	199(52.6)	
≥ 24.0	419(36.1)	60(17.1)	199(46.2)	160(42.3)	
History of smoking, n(%)	245(21.1)	66(18.8)	92(21.3)	87(23.0)	0.376
History of alcoholism, n(%)	58(5.0)	23(6.6)	17(3.9)	18(4.8)	0.243
Serum lipid-lipoprotein levels					
TC, mmol/L	4.47 ± 1.30	4.23 ± 1.15	4.56 ± 1.42	4.60 ± 1.26	< 0.001
TG, mmol/L	1.81 ± 1.63	1.33 ± 0.79	1.95 ± 1.86	2.10 ± 1.83	< 0.001
HDL-C, mmol/L	1.12 ± 0.36	1.17 ± 0.43	1.07 ± 0.32	1.12 ± 0.31	0.001
LDL-C, mmol/L	2.45 ± 0.86	2.36 ± 0.77	2.52 ± 0.90	2.46 ± 0.88	0.023
Apo-A1, g/L	1.03 ± 0.27	1.01 ± 0.32	1.01 ± 0.26	1.05 ± 0.23	0.046
Apo-B, g/L	0.80 ± 0.25	0.75 ± 0.23	0.83 ± 0.26	0.82 ± 0.26	< 0.001

T2DM, type 2 diabetes mellitus; CAD, coronary artery disease; BMI, body mass index; Values for age expressed as mean ± SD. TC, total cholesterol; TG, triglycerides; HDL-C, high-density lipoprotein-cholesterol; LDL-C, low-density lipoprotein-cholesterol; Apo-A1, apolipoprotein A1; Apo-B, apolipoprotein B

### Distribution of the *APOE* genotypes and alleles between the patients and controls

The results of Hardy-Weinberg equilibrium test showed that the *APOE* genotypes in the T2DM patients (*χ*^2^ = 5.957, *p* = 0.202), T2DM + CAD patients (*χ*^2^ = 1.825, *p* = 0.768), and controls (*χ*^2^ = 7.970, *p* = 0.093) confirmed to the Hardy-Weinberg equilibrium, respectively. Compared to the controls, the frequency of the *APOE* ɛ3/ɛ4 genotype was higher in the T2DM + CAD patients (22.0% vs. 10.4%, *p* < 0.001), and the frequency of the *APOE* ɛ3/ɛ3 genotype was lower (60.1% vs. 72.6%, *p* < 0.001). The frequency of the ε4 allele was higher (13.9% vs. 7.1%, *p* < 0.001) and ε3 allele was lower (77.4% vs. 84.3%, *p* < 0.001) in the T2DM + CAD patients than that in the controls (Table 2).

**Table 2:** Distribution frequencies of *APOE* genotype and allele in patients and controls

Variable	Genotype/allele	Total (*n* = 1160)	Controls (*n* = 351)	T2DM patients (*n* = 431)	T2DM + CAD patients (*n* = 378)	χ^2^	p values
*APOE* genotype							
	ɛ2/ɛ2	6(0.5%)	2(0.6%)	1(0.2%)	3(0.8%)	1.261	0.525
	ɛ2/ɛ3	151(13.0%)	48(13.7%)	55(12.8%)	48(12.7%)	0.193	0.904
	ɛ2/ɛ4	28(2.4%)	8(2.3%)	8(1.9%)	12(3.2%)	1.525	0.471
	ɛ3/ɛ3	800(69.0%)	255(72.6%)	318(73.8%)	227(60.1%)	20.926	< 0.001
	ɛ3/ɛ4	162(14.0%)	34(9.7%)	45(10.4%)	83(22.0%)	29.900	< 0.001
	ɛ4/ɛ4	13(1.1%)	4(1.1%)	4(0.9%)	5(1.3%)	0.285	0.939
*APOE* allele							
	ε2	191(8.2%)	60(8.5%)	65(7.5%)	66(8.7%)	0.886	0.647
	ε3	1913(82.5%)	592(84.3%)	736(85.4%)	585(77.4%)	20.271	< 0.001
	ε4	216(9.3%)	50(7.1%)	61(7.1%)	105(13.9%)	27.843	< 0.001
	HWE (χ^2^, *P*)	χ^2^ = 9.060, *p* = 0.060	χ^2^ = 7.970, *p* = 0.093	χ^2^ = 5.957, *p* = 0.202	χ^2^ = 1.825, *p* = 0.768		

HWE, Hardy Weinberg Equilibrium

### Clinical characteristics and serum lipid-lipoprotein levels of T2DM + CAD patients stratified by *APOE* ɛ2, ɛ3, ɛ4 alleles

Clinical characteristics and serum lipid-lipoprotein levels were compared among T2DM + CAD patients carried different *APOE* alleles. T2DM + CAD patients with the E2/E4 genotype (*n* = 12) were excluded from the analysis of the relationship between *APOE* alleles and clinical characteristics of patients because of the opposite effects of the ε2 and ε4 alleles in lipid metabolism [21, 24]. The T2DM + CAD patients with ɛ4 allele had higher level in LDL-C (2.70 ± 0.76 mmol/L vs. 2.21 ± 0.84 mmol/L) while had higher level in ApoB (0.87 ± 0.23 g/L vs. 0.76 ± 0.26 g/L) than those with ɛ2 allele (all *p* < 0.05). The T2DM + CAD patients with ɛ4 allele had higher level in LDL-C (2.70 ± 0.76 mmol/L vs. 2.43 ± 0.91 mmol/L) than those with ɛ3 allele (*p* < 0.05). There were no statistically significant differences in the distributions of gender, BMI, history of smoking, history of alcoholism, and the level of TC and LDL-C among T2DM + CAD patients carried *APOE* ɛ2, ɛ3 and ɛ4 alleles, respectively (Table 3).

**Table 3:** Clinical characteristics and serum lipid-lipoprotein levels of T2DM + CAD patients stratified by *APOE* ɛ2, ɛ3, ɛ4 alleles

Variables	ε2 (ɛ2/ɛ2 + ɛ2/ɛ3) (*n* = 51)	ε3 (ɛ3/ɛ3) (*n* = 227)	ε4 (ɛ3/ɛ4 + ɛ4/ɛ4) (*n* = 88)	p values
Age, years				
< 60, n(%)	10(19.6)	75(33.0)	16(18.2)	0.012 (χ^2^=8.899)
≥ 60, n(%)	41(80.4)	152(67.0)	72(81.8)	
Gender				
Male, n(%)	28(54.9)	149(65.6)	59(67.0)	0.307 (χ^2^=2.429)
Female, n(%)	23(45.1)	78(34.4)	29(33.0)	
BMI (kg/m^2^)				
< 18.5	3(5.9)	15(6.6)	1(1.1)	0.335 (χ^2^=4.519)
18.5–23.9	28(54.9)	116(51.1)	47(53.4)	
≥ 24.0	20(39.2)	96(42.3)	40(45.5)	
History of smoking, n(%)	7(13.7)	57(25.1)	20(22.7)	0.221 (χ^2^=3.056)
History of alcoholism, n(%)	0(0)	15(6.6)	3(3.4)	0.106 (χ^2^=4.156)
Serum lipid-lipoprotein levels				
TC, mmol/L	4.49 ± 1.12	4.56 ± 1.36	4.74 ± 1.09	0.435
TG, mmol/L	2.46 ± 1.60	2.11 ± 2.11	1.75 ± 0.92^#^	0.076
HDL-C, mmol/L	1.11 ± 0.29	1.11 ± 0.31	1.17 ± 0.32	0.311
LDL-C, mmol/L	2.21 ± 0.84	2.43 ± 0.91	2.70 ± 0.76^#^*	0.004
Apo-A1, g/L	1.08 ± 0.21	1.04 ± 0.24	1.07 ± 0.23	0.299
Apo-B, g/L	0.76 ± 0.26	0.82 ± 0.27	0.87 ± 0.23^#^	0.049

T2DM, type 2 diabetes mellitus; CAD, coronary artery disease; BMI, body mass index; Values for age expressed as mean ± SD. TC, total cholesterol; TG, triglycerides; HDL-C, high-density lipoprotein-cholesterol; LDL-C, low-density lipoprotein-cholesterol; Apo-A1, apolipoprotein A1; Apo-B, apolipoprotein B

^#^compared with ɛ2, *p* < 0.05;

*compared with ɛ3, *p* < 0.05

### Association of *APOE* gene polymorphisms with T2DM + CAD patients

The logistic regression analysis showed that age ≥ 60 years old (adjusted OR = 5.12, 95% CI= 3.57–7.34, *p* < 0.001), BMI ≥ 24.0 kg/m^2^ (adjusted OR = 3.42, 95% CI = 2.28–5.12, *p* < 0.001), *APOE* ɛ3/ɛ4 genotype (adjusted OR = 1.93, 95% CI = 1.18–3.14, *p* = 0.008) and ɛ4 allele (adjusted OR = 1.97, 95% CI = 1.23–3.17, *p* = 0.005) were independent risk factors for T2DM + CAD. In addition, age ≥ 60 years old (adjusted OR = 2.81, 95% CI = 2.02–3.91, *p* < 0.001), and BMI ≥ 24.0 kg/m^2^ (adjusted OR = 3.29, 95% CI = 2.28–4.75, *p* < 0.001) were independent risk factors for T2DM. However, the *APOE* genotypes and alleles were not found to have relationship with the risk of T2DM (Table 4).

**Table 4:** Logistic regression analysis of risk factors for T2DM, and T2DM + CAD.

Variables	T2DM				T2DM + CAD			
	Univariate OR (95% CI)	p values	Multivariate OR (95% CI)	p values	Univariate OR (95% CI)	p values	Multivariate OR (95% CI)	p values
Age (≥ 60/<60)	2.38 (1.78–3.19)	< 0.001	2.81 (2.02–3.91)	< 0.001	4.41 (3.21–6.05)	< 0.001	5.12 (3.57–7.34)	< 0.001
Gender (Male/Female)	0.95 (0.70–1.28)	0.721	0.91 (0.63–1.30)	0.594	0.99 (0.73–1.35)	0.946	1.03 (0.70–1.52)	0.848
BMI (kg/m^2^)								
18.5–23.9	1.00 (reference)	-	1.00 (reference)	-	1.00 (reference)	-	1.00 (reference)	-
< 18.5	0.15 (0.08–0.26)	< 0.001	0.14 (0.08–0.25)	< 0.001	0.20 (0.12–0.35)	< 0.001	0.20 (0.11–0.36)	< 0.001
≥ 24.0	2.93 (2.07–4.15)	< 0.001	3.29 (2.28–4.75)	< 0.001	2.59 (1.81–3.70)	< 0.001	3.42 (2.28–5.12)	< 0.001
History of smoking (Yes/No)	1.17 (0.82–1.67)	0.381	1.47 (0.92–2.32)	0.105	1.27 (0.87–1.83)	0.192	1.39 (0.85–2.27)	0.185
History of alcoholism (Yes/No)	0.58 (0.31–1.11)	0.100	0.66 (0.29–1.47)	0.307	0.72 (0.38–1.36)	0.310	0.92 (0.41–2.07)	0.841
*APOE* genotypes								
ɛ3/ɛ3	1.00 (reference)	-	1.00 (reference)	-	1.00 (reference)	-	1.00 (reference)	-
ɛ2/ɛ2	0.40 (0.04–4.45)	0.457	0.83 (0.06–12.48)	0.893	1.69 (0.28–10.18)	0.570	3.57 (0.28–45.37)	0.326
ɛ2/ɛ3	0.92 (0.60–1.40)	0.693	1.15 (0.70–1.87)	0.585	1.12 (0.73–1.74)	0.603	1.28 (0.75–2.16)	0.364
ɛ3/ɛ4	1.06 (0.66–1.71)	0.806	1.07 (0.63–1.81)	0.805	2.74 (1.77–4.25)	< 0.001	1.93 (1.18–3.14)	0.008
ɛ4/ɛ4	0.80 (0.20–3.24)	0.757	2.43 (0.45–13.24)	0.306	1.40 (0.37–5.29)	0.616	2.67 (0.52–13.61)	0.239
*APOE* alleles								
ε3	1.00 (reference)	-	1.00 (reference)	-	1.00 (reference)	-	1.00 (reference)	-
ε2	0.90 (0.59–1.36)	0.612	1.13 (0.70–1.84)	0.611	1.15 (0.75–1.76)	0.534	1.32 (0.79–2.22)	0.287
ε4	1.03 (0.66–1.63)	0.885	1.14 (0.68–1.90)	0.614	2.60 (1.71–3.96)	< 0.001	1.97 (1.23–3.17)	0.005

## Discussion

CVD is an important cause of morbidity and mortality in patients with T2DM, however, T2DM has a wide range of risks and does not necessarily equate to the risk of CVD, indicating the importance of risk factor identification and risk assessment in patients with T2DM [32]. T2DM is strongly associated with an increased risk of CVD, and the two separate diseases often co-exist and affect each other [33, 34]. CAD is one of the most common manifestations of cardiovascular disease in T2DM patients and is largely associated with accelerated atherosclerosis driven by inflammation [34]. Cardiovascular disease mortality and morbidity are increased in patients with diabetes compared to non-DM patients [35]. The severity of coronary heart disease was positively correlated with DM [36]. Lipid levels have been linked to the risk of CAD and T2DM, and dyslipidemia is a significant risk factor for CAD and T2DM [37, 38]. ApoE is involved in lipid metabolism, so that the *APOE* gene polymorphisms are associated with CAD and T2DM. In this study, we examined the relationship between *APOE* gene polymorphisms and CAD in T2DM patients.

*APOE* ɛ3/ɛ4 genotype and ɛ4 allele were independent risk factors for T2DM + CAD, however, *APOE* genotypes and alleles were not found to be an independent risk factor for T2DM. Wu et al. found that the *APOE* ɛ3/ɛ4 genotype has a significantly increased risk of T2DM + CAD [39]. Ozuynuk AS et al. showed that the *APOE* ε2 carriers had a lower risk for T2DM + CAD in a Turkish population [40]. Chaudhary R et al. showed that the *APOE* ε4 allele is a risk factor for CAD, T2DM, and T2DM + CAD in a study from a population in Thailand [41]. Another study showed that the *APOE* ε4 allele was associated with an increased risk of CAD in T2DM patients, while ε2 allele was not associated with CAD risk [42]. Liu et al. found that *APOE* ε4 allele was higher in T2DM + CVD patients compared with controls [43]. In the Iranian population, T2DM patients with the *APOE* ε2 and ε4 alleles have a higher risk of developing CAD than non-DM patients [44].

In addition, the *APOE* genotypes and alleles were not found to have relationship with the risk of T2DM. Several studies have reported the relationship between *APOE* polymorphism and impaired glucose metabolism and the risk of DM. Zeng et al. found that *APOE* ɛ3/ɛ4 genotype and ε4 allele are associated with an increased risk of T2DM among a Han Chinese population [22]. The *APOE* ɛ4 allele may be a risk factor for T2DM [45]. A meta-analysis suggests that the E2 allele of *APOE* gene may be a risk factor for T2DM [46]. Another meta-analysis based on a Chinese Han population suggests that *APOE* ε2 and ε4 alleles may be associated with an increased risk of T2DM [47]. On the other hand, some studies have come to negative or even contrary conclusions. Chatinun Srirojnopkun et al. found no association between *APOE* polymorphisms and T2DM [48]. In addition, studies have found that ɛ4 carriers are less likely to develop T2DM [49, 50]. Cátia Santos-Ferreira et al. showed that a significant increase in T2DM incidence in *APOE* ɛ2 carriers [51].

In this study, age ≥ 60 years old and BMI ≥ 24.0 kg/m^2^ were independent risk factors for T2DM and T2DM + CAD. In other words, older and overweight persons are more likely to have T2DM and T2DM + CAD. The causal relationship between being overweight or obesity and T2DM and CAD has been widely recognized and supported [52, 53]. People with a higher BMI are more likely to develop DM and CAD [36, 54]. A study from Iran showed that overweight and smoking are the most common risk factors for CAD [55]. Saintrain MVL et al. found that patients with a BMI of 18.6 to 24.9 km/m^2^ are more at risk of malnutrition, and nutritional status is associated with CAD [56]. Bai et al. suggested that BMI had the strongest association with T2DM in women [57]. In addition, some studies have found that smoking and old age are factors that increase the risk of developing T2DM [58]. A report from the United States showed that being male, older, and having a history of smoking were significantly associated with CAD in T2DM patients [59]. T2DM + CAD patients tend to be older [60].

As for the role of APOE in the development of CAD in DM patients, it may be related to inflammatory response, oxidative stress, insulin resistance and other mechanisms. Chronic inflammation and oxidative stress exist in CAD, and subclinical inflammation and insulin resistance exist in DM patients [61, 62]. First, the normal vascular endothelial system can maintain the properties of anti-adhesion, regulate the contractile tension of blood vessels and affect coagulation. However, the high concentration of blood glucose and free fatty acids in the blood of DM patients can induce the occurrence of vascular wall inflammation [63], destroy the normal function of endothelial cells, and lead to the excessive secretion of inflammatory factors and chemokines by immune cells, causing systemic inflammation [64]. Endothelial cell dysfunction can disrupt vascular balance, promote inflammation and thrombosis, and ultimately increase the risk of CVD. Studies have shown that ApoE-/- mice can develop severe leukocytosis and lack a cholesterol efflux mechanism, and their accumulated cholesterol can lead to an inflammatory response [65, 66]. Second, insulin resistance is the body’s weakened response to endogenous or exogenous insulin, which is related to the disorder of lipid metabolism. Reduced adiponectin levels can lead to insulin resistance, which is involved in the occurrence of DM and CAD [67]. In T2DM, adipocytes show insulin resistance, fat continues to dissolve, and free fatty acids are elevated and transported to the liver, leading to an increase in hepatogenic very low density lipoprotein cholesterol, which ultimately leads to increased atherosclerosis and further promotes the occurrence of CAD [68]. And experimental studies in animals have shown that ApoE may be related to insulin secretion [69]. Third, the disorder of pro-oxidation factors and antioxidant factors can lead to oxidative stress [70]. Reactive oxygen species (ROS) play an important role in vascular intimal thickening, vascular remodeling and arterial vascular injury [71]. Of course, more researches are needed to uncover the exact mechanism.

### Limitations

In this study, *APOE* ɛ3/ɛ4 genotype and ɛ4 allele were independent risk factors for T2DM + CAD, but not for T2DM. It suggests that T2DM patients with *APOE* ɛ3/ɛ4 genotype or ɛ4 allele need to be monitored for CAD risk. Some deficiencies in this study should be pointed out. First of all, this case-control study is based on hospitalized patients and physical examination subjects, and selection bias of population is inevitable. Secondly, the overweight population in this study was simply classified according to the BMI standard of Chinese people, the BMI cutoff values for T2DM and T2DM + CAD were not analyzed and determined. Confirmation of a race-specific BMI cutoff can optimize prevention, early diagnosis, and timely management of T2DM [72, 73]. Thirdly, the relationship between *APOE* gene polymorphism and the therapeutic effect of medication was not analyzed.

### Future directions

More and more in-depth studies are needed to clarify the relationship between *APOE* polymorphisms and the risk of T2DM and T2DM + CAD, as well as the mechanism of action. With the rapid development of molecular biology technology, the genetic detection of chronic diseases has become possible. While detecting genetic variation is simple, explaining the role of these genetic polymorphisms is critical and not easy. Although genetic risk tests for T2DM + CAD can provide a reference for clinicians in diagnosis and treatment, their current clinical application is limited. There are also deficiencies in the ability of genetic counseling and the lack of knowledge about the use of genetic testing among clinicians. These are the issues we need to pay attention to in the future.

## Conclusion

In summary, *APOE* ɛ3/ɛ4 genotype and ɛ4 allele maybe independent risk factors for T2DM complicated with CAD. In other words, elderly, overweight T2DM patients who carried the *APOE* ɛ3/ɛ4 genotype and ɛ4 allele need to be aware of the risk of CAD.

## Data Availability

No datasets were generated or analysed during the current study.
